# Incidental metastatic mediastinal atypical carcinoid in a patient with parathyroid adenoma: a case report

**DOI:** 10.1186/s13256-017-1234-2

**Published:** 2017-03-26

**Authors:** Zareen Kiran, Asma Ahmed, Owais Rashid, Saira Fatima, Faizan Malik, Saulat Fatimi, Mubassher Ikram

**Affiliations:** 1 Section of Endocrinology, Department of Medicine, Aga Khan University Hospital, Stadium Road, Karachi, Pakistan; 2 Department of Histopathology, Aga Khan University Hospital, Stadium Road, Karachi, Pakistan; 3 Department of Cardiothoracic Surgery, Aga Khan University Hospital, Stadium Road, Karachi, Pakistan; 4 Department of Ear, Nose & Throat, Aga Khan University Hospital, Stadium Road, Karachi, Pakistan

**Keywords:** Parathyroid neoplasms, Atypical carcinoid, Multiple endocrine neoplasia, Case report

## Abstract

**Background:**

Atypical carcinoid arising from the mediastinal tissue is a rare neuroendocrine tumor and an association with parathyroid adenoma is very unusual. We report an unusual case of atypical carcinoid of mediastinum with metastasis in a patient presenting with parathyroid adenoma, which is the first case to be reported from Pakistan.

**Case presentation:**

A 51-year-old Pakistani man was seen in postoperative intensive care after right parathyroidectomy and mediastinal mass resection for the management of postoperative hypocalcaemia. He had a background history of dyspnea. Examination was unremarkable. Preoperative laboratory evaluation revealed a calcium level of 12.7 mg/dl, phosphate of 1.9 mg/dl, serum albumin of 4.8 g/dl, alkaline phosphate of 94 U/L, and serum intact parathyroid hormone level 413.8 pg/ml. A technetium-99m sestamibi parathyroid scan showed right parathyroid increased tracer uptake. Further workup revealed a large mediastinal mass which was diagnosed as atypical carcinoid after Tru-Cut biopsy. He underwent right-sided parathyroidectomy and resection of the mediastinal mass. The histopathology confirmed it to be a parathyroid adenoma and atypical carcinoid tumor of his mediastinum with metastasis in his lymph node and parathyroid gland. Somatostatin receptor scintigraphy revealed a well-defined focus in his left hypochondriac region consistent with a somatostatin receptor scintigraphy-avid tumor. He was started on everolimus and planned for octreotide therapy.

**Conclusions:**

We describe an incidental finding of atypical carcinoid of the mediastinum in a patient diagnosed as having parathyroid adenoma. Clinical manifestations of neuroendocrine syndromes are challenging. Some tumors cluster in a non-classic description with other common neoplasms. They rarely present in isolation, remain clinically silent, and need aggressive workup with the aid of imaging and histopathology.

## Background

Primary neuroendocrine tumors of the mediastinum are rare. They can arise from thymic or non-thymic tissue, can be low to high grade and classified as well-differentiated or poorly differentiated neoplasms [1]. Among the well-differentiated category, we still find the terms “carcinoid” and “atypical” carcinoid in use in the literature [2, 3]. Atypical thymic carcinoid, a rare type of neuroendocrine tumor, has been described as a separate entity [4] as well as in combination with multiple endocrine neoplasia (MEN) type 1 [5]; however, atypical carcinoid arising in the mediastinum is very rare [1]. Most of the literature on neuroendocrine tumors is described in isolation and no substantial data describe an association with parathyroid adenoma from the South Asian region [6, 7]. We describe an incidentally diagnosed metastatic atypical carcinoid from mediastinal tissue in a patient presenting with parathyroid adenoma, which to the best of our knowledge is the first case reported from Pakistan.

## Case presentation

**Fig. 1:**
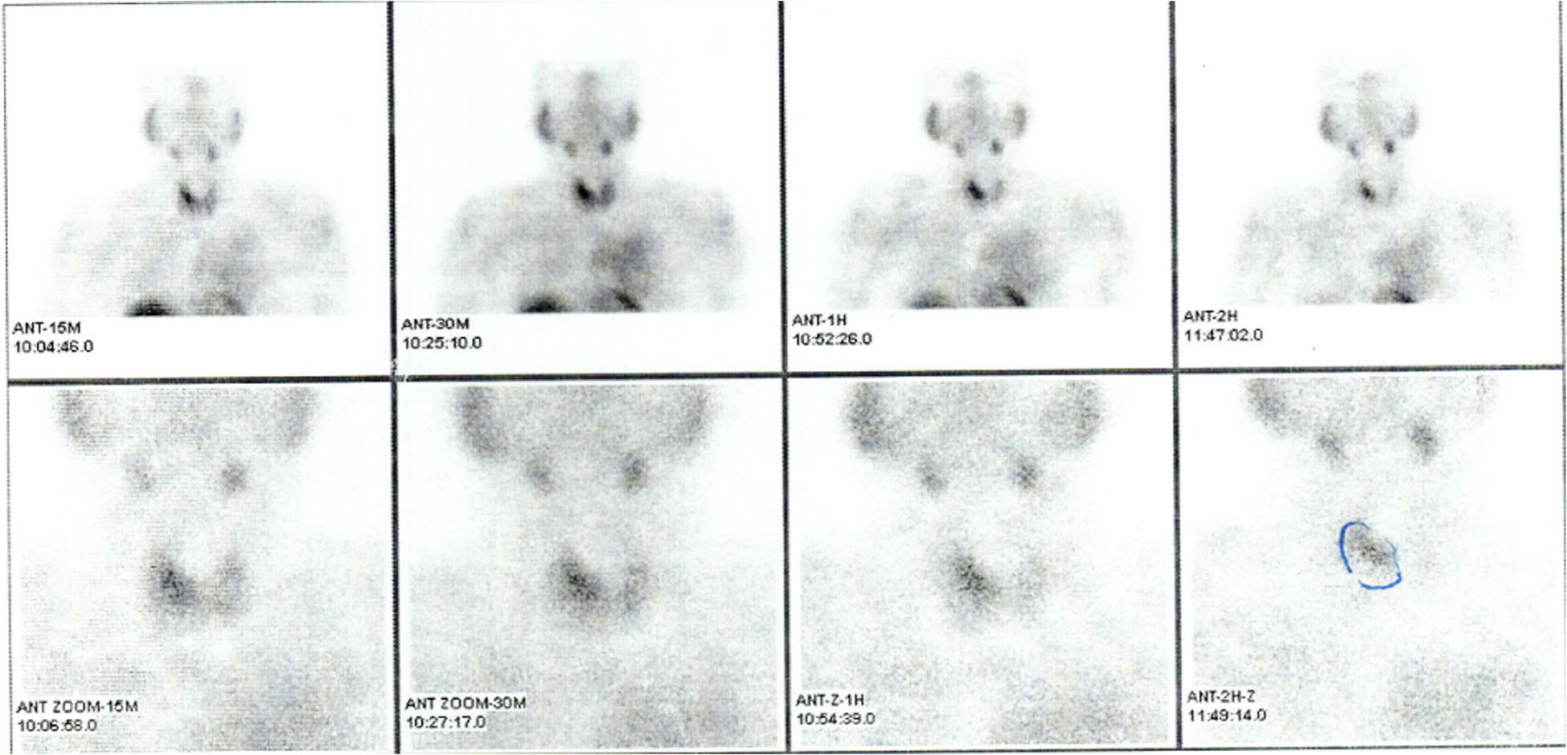
Technetium-99m sestamibi parathyroid scan. The scan shows focal area of increased uptake in right thyroid lobe

**Fig. 2:**
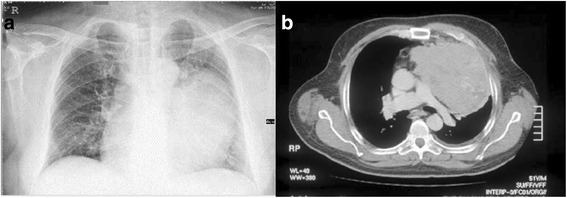
Radiological findings of mediastinal mass. **a** Chest X-ray showing large left mediastinal mass. **b** Computed tomography of chest with contrast showing left-sided radiodense mass with calcifications

A 51-year-old Pakistani man with diabetes was seen in postoperative care for hypocalcemia following parathyroidectomy of his parathyroid adenoma. He was a non-tobacco smoker, with a history of bilateral pedal edema for 1.5 months, and myalgia and dyspnea for less than 1 month. There was a history of renal stones treated with lithotripsy 7 years earlier. His medications included Mixtard-30 (human insulin), sitagliptin, metformin, and furosemide. His family history was insignificant. On examination he was well oriented and his vital signs were stable. A general physical examination revealed only mild pedal edema. A systemic examination was unremarkable. Preoperative investigations showed a calcium (Ca) level of 12.7 mg/dl, phosphate (PO_4_) 1.9 mg/dl, and serum albumin of 4.8g/dl; his alkaline phosphate 94 U/L and serum intact parathyroid hormone (PTH) level was 413.8 pg/ml. His serum creatinine was 1.04 mg/dl and he had an estimated glomerular filtration rate (eGFR) of 75.28 ml per minute/1.73m^2^. An ultrasound of his abdomen showed bilateral medullary nephrocalcinosis. An ultrasound of his neck revealed that his right thyroid lobe was mildly enlarged and both lobes showed a few small scattered solid nodules less than 10 mm. His right inferior parathyroid gland was markedly enlarged measuring 3.9×1.3×2.3 cm. Thyroid function tests were normal. A technetium-99m (^99m^Tc) sestamibi parathyroid scan was carried out, which showed a focal area of intensely increased tracer uptake in his right thyroid lobe, which after washout still showed intense tracer accumulation with a probability of parathyroid adenoma or hyperplasia in the right group of parathyroid glands (Fig. 1). On further workup, a chest X-ray revealed a left-sided mediastinal mass (Fig. 2a). Computed tomography of his chest with contrast showed an anterior mediastinal mass measuring 142 mm in diameter showing calcification (Fig. 2b). He underwent Tru-Cut biopsy of this mass and histopathology revealed fibrocollagenous tissue showing sheets of neoplastic cells. After immunohistochemical staining and based on raised proliferative index (Ki-67 10%), this tumor was classified as atypical carcinoid tumor. He was planned for resection of this tumor as well as parathyroidectomy in a one-stage surgery.

**Fig. 3:**
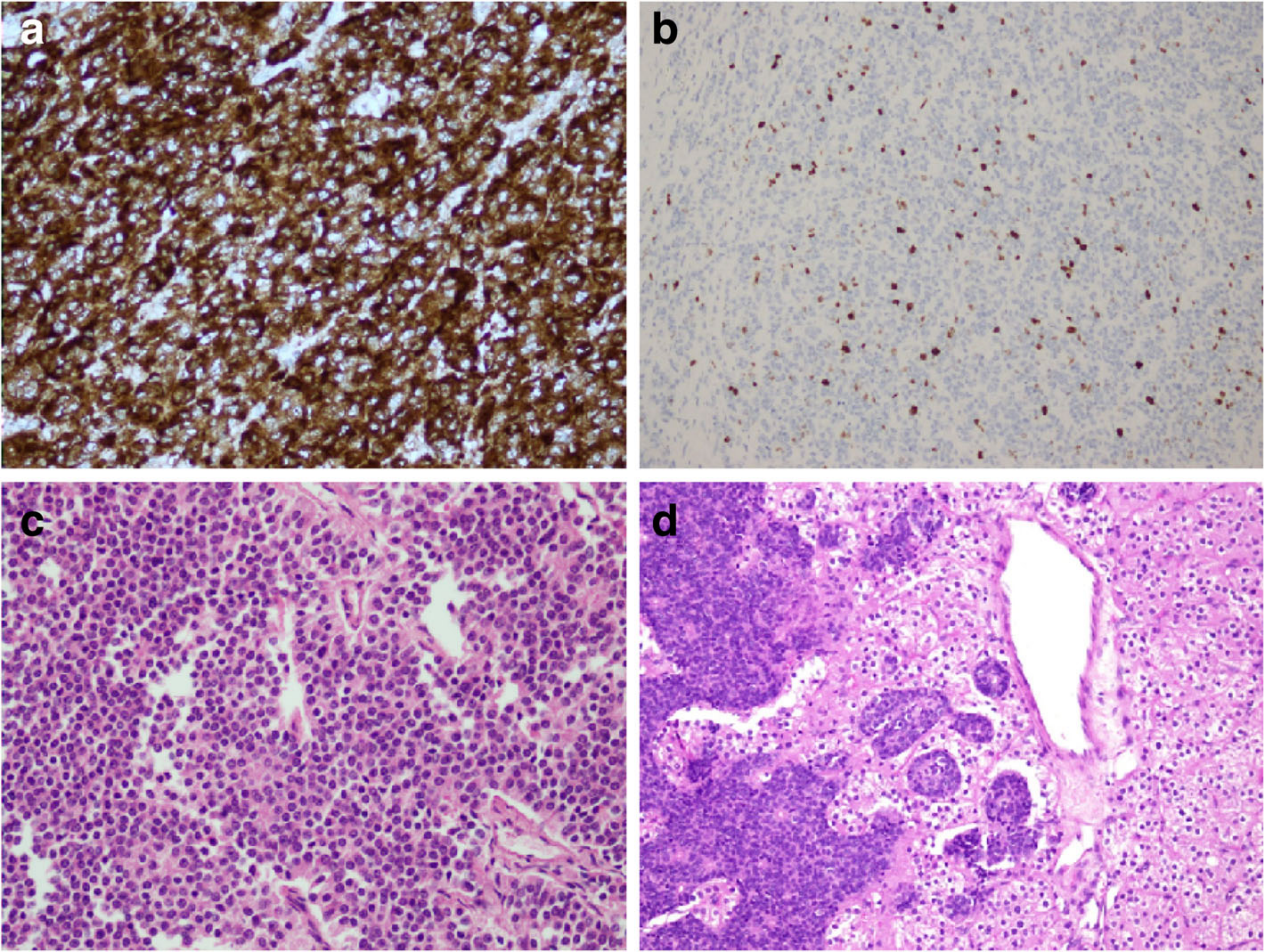
Histopathology of mediastinal mass and parathyroid tissue. **a** Immunohistochemical stain positive for chromogranin-A. **b** Mib-1 (Ki-67) positivity in mediastinal mass. **c** Hematoxylin and eosin staining of the mediastinal mass showing diffuse sheets and nests of neoplastic cells, separated by thick and thin fibrous bands. **d** Histologic findings of the parathyroid tissue without cytological atypia or mitotic activity favoring parathyroid adenoma

**Fig. 4:**
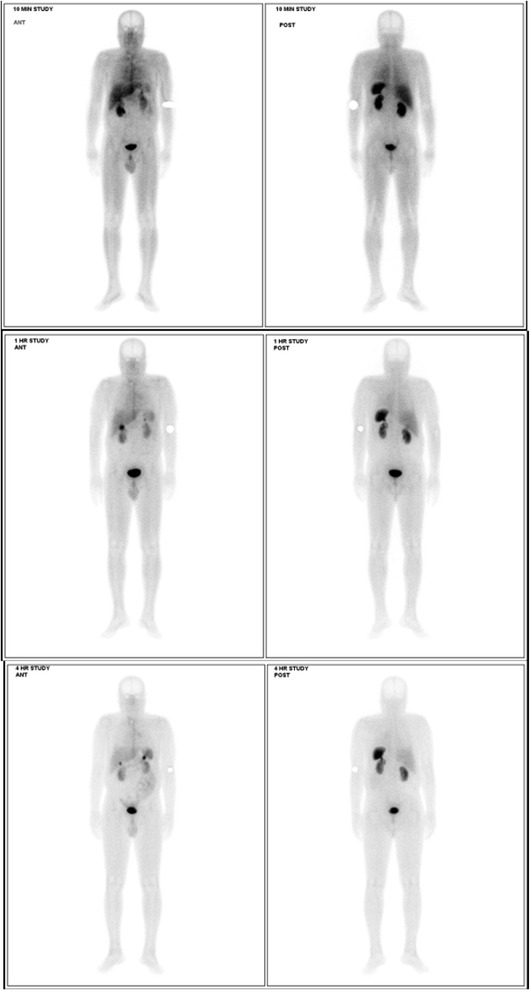
Somatostatin receptor imaging with technetium-99m hydrazinonicotinamide octreotide. Somatostatin receptor scintigraphy image revealed a well-defined focus in left hypochondrium consistent with somatostatin receptor scintigraphy-avid tumor. Another area of increased tracer uptake of moderate intensity involved the right thyroid bed

He developed hypocalcaemia postoperatively which was adequately treated. His postoperative PTH came down to 45.90 pg/ml. The histopathology of the mediastinal tumor measuring 18×15×14 cm and level 5 mediastinal lymph node measuring 0.5×0.3 cm retrieved during surgery revealed an encapsulated lesion comprising diffuse sheets and nests of neoplastic cells, separated by thick and thin fibrous bands. An occasional mitotic figure was seen: up to 2/10 to 3/10 high-power field (HPF). In some areas the tumor cells formed rosette-like structures. Large areas of necrosis were seen along with extensive homogenous, fibrotic areas (Fig. 3c). These areas were negative for Congo red or Sirius red special stains. An immunohistochemical examination showed the same reactivity pattern as the Tru-Cut biopsy. Histopathology of the parathyroid tissue showed fragmented tissue, without cytological atypia, mitotic activity, perineural invasion, indicating parathyroid adenoma (Fig. 3d). There were clusters of neuroendocrine cells, suggestive of involvement by mediastinal atypical carcinoid. A section from his lymph node was also positive for tumor metastasis. These features were consistent with atypical carcinoid tumor with lymph node metastasis. His urinary 5-hydroxyindoleacetic acid (5-HIAA) was only mildly elevated: 7.9 mg/24 hours (2–7). His serum prolactin level was 5.90 U/L (<23.8). Later, somatostatin receptor scintigraphy (SRS) revealed a well-defined focus in his left hypochondriac region between the inner border of his spleen and his left upper renal pole, consistent with SRS-avid tumor (Fig. 4). Another area of increased tracer uptake was noted involving his right thyroid bed. He has been started on everolimus and planned for octreotide therapy. He is currently asymptomatic and recovering well.

## Discussion

Neuroendocrine tumors arising from the thymus itself account for just 2 to 5% of primary thymic malignancies [8]. Atypical carcinoid is even a rarer type of neuroendocrine tumor [3]. They mostly remain asymptomatic until they metastasize at the time of presentation (66% to lymph nodes from pulmonary origin) and behave as aggressive tumors rather than slow growing carcinoids. They are different from carcinoid tumors as they mostly arise in the foregut tissues such as bronchial epithelium and thymus gland. Patients usually have normal levels of serotonin and chromogranin-A in their blood and normal levels of 5-HIAA in their urine; however, they have raised levels of serotonin and 5-hydroxytryptophan (5-HTP) in their urine [1, 3, 9].

There is a case report of atypical carcinoid originating from the thymus gland in the form of a mediastinal mass in association with hypercalcemia which was found to be due to parathyroid adenoma [4]. Another case report of MEN 1 has shown ectopic parathyroid tissue in the mediastinal mass [10] and another has reported thymic carcinoid with atypical course in such patients [11]; however, very limited data are reported on the concomitant presence of parathyroid adenoma and asymptomatic metastatic mediastinal atypical carcinoid other than thymus. A single study has described a clear distinction between such a neuroendocrine tumor originating as ectopic tissue in the mediastinum rather than from a thymus gland [1]. Our patient’s histopathology did not reveal any thymic tissue and has characteristic immunohistochemical staining for the neuroendocrine neoplasms, more specifically for atypical carcinoid tumor. The presence of sheets of cells in the parathyroid tissue (Fig. 3) of our patient presents the metastasis of atypical carcinoid tumor from the mediastinal ectopic neuroendocrine cells, which is a rare entity that has not been described before. Whether this can be the cause of parathyroid adenoma and hyperparathyroidism in our patient and what genetics could be involved is not much known. Besides parathyroid gland, it has also metastasized to lymph nodes, thyroid areas, and peripancreatic areas. Our patient had no clinical stigmata of carcinoid syndrome and has not had any airway obstructive disease (in view of large mediastinal mass). Further evaluation for pituitary tumor in the form of imaging was not possible due to financial limitations; besides, he did not have any clinical features of Cushing’s syndrome or acromegaly. Thus, our patient had multiple endocrine disorders with two neoplasms, of which one was aggressive with a guarded prognosis. Such a clustering can be very well explained as a variant of MEN syndromes and needs genetic analysis, which is not available in our region. Moreover, apart from everolimus therapy, which has been shown to improve prognosis in a trial [12], the octreotide avidity of the metastasis in our patient means that our patient needs octreotide treatment as part of ongoing care, which has limitations in a resource-poor country.

## Conclusions

We presented a case of metastatic mediastinal atypical carcinoid in a patient with parathyroid adenoma, diagnosed incidentally during preoperative workup. The unusual clustering of neuroendocrine tumors is being increasingly reported in the literature, and patients with hyperparathyroidism should be thoroughly investigated to diagnose and treat other related tumors particularly atypical carcinoid of mediastinum as it has a poor outcome and limited treatment options.

## Abbreviations

5-HIAA: 5-hydroxyindoleacetic acid
5-HTP: 5-hydroxytryptophan
Tc: Technetium-99m
Ca: Calcium
eGFR: Estimated glomerular filtration rate
HPF: High-power field
MEN: Multiple endocrine neoplasia
PO_4_: Phosphate
PTH: Parathyroid hormone
SRS: Somatostatin receptor scintigraphy
